# Supplementary material for: A Likelihood Approach for Real-Time Calibration of Stochastic Compartmental Epidemic Models
Source: PLoS Comput Biol. 2017 Jan 17;13(1):e1005257. doi: 10.1371/journal.pcbi.1005257 (PMC5240920; doi:10.1371/journal.pcbi.1005257)
Supplement: S1 File — (TAR.GZ) [file pcbi.1005257.s014.tar.gz › HSPH_Online-SI-Revision/MSS10/n10-mild/n10-mild_NEW_table0.pdf]

1	R0	{1.25046, 6.80278, 0.1}	{1.08961, 2.32578, 0.02}	{{1.06738, 2.19254, 0.}, {1.22544, 3.07157, 0.}}	{12.8701, 65.8113, 116.}	inf	157.	72.1607	{27.8266, 116.041}	54.0378	0.412062	newly	174.	191.55	{70., 317.}	44.5575	0.225499	Reff	1.06127	0.933915	{0.910932, 1.04202}	12.0079	ar	3516.	2057.3	{1491., 2669.}	41.7196
2	R0	{1.32013, 4.45847, 0.1}	{1.26136, 3.42652, 0.02}	{{1.22544, 3.07157, 0.}, {1.28596, 3.98513, 0.}}	{4.57811, 23.6138, 116.}	inf	321.	240.069	{180.171, 318.825}	26.3975	0.142918	newly	488.	452.17	{335., 570.}	15.6865	0.0751109	Reff	0.980989	0.947454	{0.927428, 0.962154}	3.53176	ar	4420.	3881.75	{3494., 4203.}	12.536
3	R0	{1.31785, 5.36796, 1.}	{1.24507, 2.98653, 0.}	{{1.22544, 2.5364, 0.}, {1.25474, 3.26445, 0.}}	{5.52284, 44.3638, 100.}	inf	363.	217.188	{149.033, 287.466}	40.1686	0.242074	newly	488.	473.66	{347., 613.}	18.3607	0.0873762	Reff	1.00407	0.966769	{0.948575, 0.979337}	3.71495	ar	4942.	3663.12	{3251., 4022.}	25.8778
4	R0	{1.38801, 6.16031, 0.1}	{1.24735, 2.63062, 0.}	{{1.24751, 2.5364, 0.}, {1.24751, 3.07157, 0.}}	{10.1337, 57.2974, 100.}	inf	457.	195.944	{118.905, 270.722}	57.1238	0.385409	newly	527.	464.97	{288., 621.}	21.4858	0.108497	Reff	1.01047	0.940635	{0.931428, 0.950321}	6.91121	ar	4790.	3644.16	{3270., 4035.}	23.9215
5	R0	{1.27955, 11.57, 1.}	{1.17044, 5.77052, 0.99}	{{1.17082, 5.77111, 1.}, {1.17082, 5.77111, 1.}}	{8.5273, 50.1251, 1.}	inf	179.	98.4243	{77.9086, 117.086}	45.0144	0.265464	newly	120.	118.59	{88., 139.}	12.5083	0.0567658	Reff	1.07866	0.996111	{0.994088, 0.998721}	7.65296	ar	3460.	2595.61	{2193., 2960.}	24.9824
6	R0	{1.22184, 4.34395, 1.}	{1.23977, 3.24383, 0.05}	{{1.22544, 3.07157, 0.}, {1.28596, 3.98513, 0.}}	{1.55168, 25.3253, 95.}	inf	231.	161.37	{88.9776, 234.944}	33.448	0.209037	newly	353.	348.83	{182., 494.}	28.2011	0.141009	Reff	1.01352	1.03697	{1.01786, 1.06195}	2.3541	ar	3524.	3379.46	{2804., 3896.}	10.2866
7	R0	{1.23856, 4.59068, 0.1}	{1.24047, 3.0422, 0.01}	{{1.22544, 2.5364, 0.}, {1.25474, 3.26445, 0.}}	{1.15122, 33.731, 108.}	inf	200.	146.569	{86.5195, 215.69}	31.2977	0.185463	newly	288.	326.77	{183., 469.}	33.1979	0.136171	Reff	0.970659	0.978772	{0.96119, 0.994241}	1.26694	ar	3855.	3338.86	{2826., 3822.}	14.2112
8	R0	{1.34531, 10.6689, 1.}	{1.23146, 3.31838, 0.3}	{{1.21159, 3.07157, 0.}, {1.25474, 3.57392, 1.}}	{8.46243, 68.8967, 72.}	inf	360.	147.429	{81.6118, 197.891}	59.0475	0.413357	newly	242.	299.25	{169., 406.}	36.5909	0.140704	Reff	1.0562	0.992977	{0.975142, 1.0207}	5.98613	ar	4852.	3223.16	{2808., 3696.}	33.5705
9	R0	{1.31775, 5.19226, 0.1}	{1.25936, 3.06386, 0.06}	{{1.24751, 2.5364, 0.}, {1.28596, 3.98513, 0.}}	{4.56491, 40.9917, 152.}	inf	400.	231.38	{132.417, 320.233}	42.155	0.262812	newly	527.	500.21	{326., 644.}	19.2543	0.0921469	Reff	1.00689	0.983405	{0.970179, 0.997974}	2.50107	ar	4708.	3764.28	{3259., 4246.}	20.2736
10	R0	{1.25916, 6.89207, 0.1}	{1.227, 3.12588, 0.12}	{{1.21159, 3.07157, 0.}, {1.24751, 3.57392, 1.}}	{2.55384, 54.6453, 196.}	inf	255.	118.421	{65.8704, 186.468}	53.5605	0.368949	newly	246.	252.97	{132., 394.}	29.4106	0.140156	Reff	0.978871	0.97063	{0.959886, 0.988343}	1.15146	ar	3823.	3152.86	{2605., 3563.}	17.6396
11	R0	{1.20377, 5.46566, 0.1}	{1.07226, 1.82968, 1.98}	{{1.06836, 1.81005, 2.}, {1.06836, 1.81005, 2.}}	{11.0338, 66.5241, 1884.}	inf	165.	76.2664	{27.1546, 131.889}	53.8835	0.419661	newly	221.	235.97	{77., 404.}	44.4932	0.221846	Reff	0.992749	0.893791	{0.884698, 0.896026}	10.106	ar	3114.	2141.61	{1715., 2457.}	31.3574
12	R0	{1.27878, 2.55804, 0.1}	{1.24751, 2.5364, 0.}	{{1.24751, 2.5364, 0.}, {1.24751, 2.5364, 0.}}	{2.44507, 0.845784, 100.}	inf	209.	226.561	{150.533, 305.935}	24.6563	0.113946	newly	522.	537.06	{302., 697.}	21.8774	0.107345	Reff	0.948983	0.923589	{0.913365, 0.932777}	2.67592	ar	3930.	3757.29	{3254., 4096.}	6.9631
13	R0	{1.41227, 5.45696, 0.1}	{1.40042, 5.69019, 0.96}	{{1.40191, 5.7404, 1.}, {1.40191, 5.7404, 1.}}	{1.02381, 5.67244, 868.}	inf	402.	433.28	{372.083, 485.114}	11.1741	0.0458285	newly	505.	525.5	{453., 589.}	9.23168	0.039044	Reff	1.01556	1.00263	{0.992931, 1.01253}	1.36372	ar	4822.	5052.61	{4741., 5301.}	5.56802
14	R0	{1.32917, 7.27593, 1.}	{1.35277, 6.73575, 1.93}	{{1.28596, 5.40945, 2.}, {1.36632, 6.88118, 2.}}	{2.91374, 13.5937, 105.}	inf	374.	370.676	{264.677, 426.569}	14.8296	0.0707222	newly	351.	379.37	{312., 434.}	13.4672	0.0560612	Reff	0.994485	1.01203	{0.98586, 1.0199}	2.40691	ar	4758.	4662.71	{4174., 4964.}	6.39996
15	R0	{1.31629, 9.97481, 2.}	{1.22649, 3.36013, 0.4}	{{1.21159, 3.07157, 0.}, {1.25474, 3.57392, 1.}}	{6.82247, 66.3138, 80.}	inf	325.	128.026	{71.006, 186.229}	60.6073	0.435964	newly	238.	274.83	{154., 387.}	34.7101	0.141552	Reff	1.102	1.05096	{1.03191, 1.08284}	4.65087	ar	4543.	3183.87	{2588., 3733.}	29.917
16	R0	{1.36735, 5.74166, 0.1}	{1.25482, 3.34013, 0.02}	{{1.22544, 2.5364, 0.}, {1.28596, 3.98513, 0.}}	{8.23011, 41.8264, 118.}	inf	345.	214.286	{130.904, 279.978}	37.888	0.22523	newly	435.	425.52	{288., 542.}	17.2092	0.0794957	Reff	1.05464	0.984188	{0.959653, 1.00734}	6.67995	ar	4516.	3694.88	{3216., 4135.}	18.1882
17	R0	{1.27524, 13.2396, 0.1}	{1.83611, 18.849, 1.}	{{1.80508, 18.4377, 1.}, {1.86254, 19.1994, 1.}}	{43.9817, 42.3684, 900.}	inf	189.	322.406	{252.084, 387.263}	70.5854	0.225439	newly	111.	136.05	{108., 167.}	24.7658	0.0920515	Reff	1.19643	1.698	{1.67679, 1.71747}	41.922	ar	3731.	7439.54	{7240., 7613.}	99.398
18	R0	{1.21884, 7.71545, 1.}	{1.20963, 3.43214, 0.76}	{{1.21159, 3.07157, 0.}, {1.22544, 3.57392, 1.}}	{1.08517, 55.516, 28.}	inf	231.	115.083	{70.7714, 152.652}	50.1806	0.326391	newly	192.	236.31	{145., 335.}	35.4323	0.137528	Reff	1.02078	1.02706	{1.02219, 1.0418}	1.47225	ar	3080.	2978.9	{2232., 3509.}	12.6078
19	R0	{1.34879, 6.71464, 0.1}	{1.24751, 2.5364, 0.}	{{1.24751, 2.5364, 0.}, {1.24751, 2.5364, 0.}}	{7.50873, 62.2258, 100.}	inf	378.	148.8	{84.3797, 203.513}	60.6349	0.425521	newly	370.	341.13	{197., 451.}	22.2027	0.11011	Reff	0.926888	0.885884	{0.878991, 0.892817}	4.42389	ar	4585.	3653.33	{3304., 3917.}	20.32
20	R0	{1.37284, 5.75036, 0.1}	{1.24605, 2.64081, 0.}	{{1.22544, 2.5364, 0.}, {1.24751, 3.07157, 0.}}	{9.23552, 54.0757, 100.}	inf	291.	161.751	{87.8868, 224.671}	44.4154	0.280347	newly	356.	399.13	{220., 566.}	30.5758	0.127927	Reff	1.04981	0.968959	{0.956807, 0.979312}	7.70158	ar	4750.	3456.42	{2972., 3906.}	27.2333
21	R0	{1.29938, 5.36419, 0.1}	{1.24469, 2.94485, 0.}	{{1.22544, 2.5364, 0.}, {1.25474, 3.26445, 0.}}	{4.20917, 45.1017, 100.}	inf	347.	203.169	{128.188, 271.215}	41.45	0.250995	newly	456.	441.53	{284., 609.}	20.2083	0.0952383	Reff	0.968298	0.945437	{0.928128, 0.958332}	2.36096	ar	4337.	3647.74	{3259., 4014.}	16.0032
22	R0	{1.31882, 7.47626, 0.1}	{1.22563, 3.2492, 0.3}	{{1.21159, 3.07157, 0.}, {1.25474, 3.57392, 1.}}	{7.06644, 56.5397, 340.}	inf	278.	132.093	{68.1089, 183.64}	52.4847	0.349715	newly	256.	275.06	{150., 398.}	28.0234	0.121944	Reff	1.04292	0.98711	{0.97244, 1.00768}	5.35157	ar	3977.	3169.48	{2720., 3513.}	20.3555
23	R0	{1.26215, 3.46375, 0.1}	{1.23953, 2.94661, 0.}	{{1.22544, 2.5364, 0.}, {1.25474, 3.26445, 0.}}	{1.79188, 14.9301, 100.}	inf	212.	169.121	{88.2711, 235.82}	26.5304	0.158866	newly	394.	372.38	{224., 507.}	24.0863	0.123861	Reff	0.984098	0.971791	{0.954822, 0.987701}	1.46232	ar	3607.	3372.25	{2813., 3805.}	9.76296
24	R0	{1.16712, 4.19464, 1.}	{1.13169, 2.08821, 1.31}	{{1.06738, 1.81005, 0.}, {1.24751, 2.5364, 3.}}	{7.28619, 50.2171, 117.}	inf	126.	73.3192	{23.551, 118.862}	44.7549	0.315907	newly	188.	210.05	{69., 351.}	45.367	0.206612	Reff	0.946418	0.923567	{0.869471, 1.01843}	7.03679	ar	3000.	2495.64	{1932., 3231.}	21.0253
25	R0	{1.22751, 3.34517, 0.1}	{1.84146, 9.17878, 1.07}	{{1.24751, 2.5364, 0.}, {2.29887, 14.662, 2.}}	{50.0156, 200.268, 1066.}	inf	170.	706.283	{135.001, 1160.87}	326.346	0.508276	newly	366.	635.45	{373., 769.}	78.123	0.239904	Reff	1.07714	1.48761	{1.08722, 1.81504}	38.1071	ar	3086.	6381.27	{3409., 8721.}	106.939
26	R0	{1.18747, 5.88083, 1.}	{1.2439, 2.64194, 0.}	{{1.22544, 2.5364, 0.}, {1.24751, 3.07157, 0.}}	{4.75251, 55.0753, 100.}	inf	219.	107.079	{58.9547, 154.641}	51.1056	0.348843	newly	259.	259.94	{146., 365.}	28.3552	0.141934	Reff	0.891909	0.948224	{0.932988, 0.956996}	6.31405	ar	3614.	3232.15	{2845., 3584.}	11.2936
27	R0	{1.29023, 4.76202, 0.1}	{1.24806, 2.57073, 0.}	{{1.24751, 2.5364, 0.}, {1.24751, 2.5364, 0.}}	{3.26832, 46.016, 100.}	inf	314.	172.235	{102.99, 237.743}	45.148	0.285491	newly	412.	413.2	{252., 570.}	23.9515	0.112976	Reff	0.948061	0.934764	{0.926186, 0.943027}	1.44195	ar	4245.	3574.91	{3098., 3944.}	15.8466
28	R0	{1.2258, 12.9056, 3.}	{1.13916, 4.44032, 1.59}	{{1.11523, 3.74835, 1.}, {1.17082, 5.77111, 2.}}	{7.06787, 65.5938, 47.}	inf	204.	69.0808	{32.2826, 104.706}	66.1369	0.507986	newly	111.	100.74	{52., 144.}	26.4324	0.137733	Reff	0.977943	0.924158	{0.903341, 0.949699}	5.57081	ar	3639.	2440.61	{2062., 2899.}	32.9318
29	R0	{1.34702, 6.29049, 0.1}	{1.24787, 3.28449, 0.03}	{{1.22544, 3.07157, 0.}, {1.28596, 3.98513, 0.}}	{7.3609, 47.7864, 126.}	inf	319.	184.209	{128.498, 241.878}	42.2542	0.256826	newly	360.	360.61	{255., 465.}	19.8861	0.0924092	Reff	0.99693	0.940337	{0.919819, 0.963046}	5.6767	ar	4560.	3605.8	{3226., 4025.}	20.9254
30	R0	{1.37931, 6.78741, 1.}	{1.25891, 3.43986, 0.}	{{1.22544, 3.07157, 0.}, {1.28596, 3.98513, 0.}}	{8.7289, 49.3201, 100.}	inf	364.	167.057	{100.259, 221.297}	54.1051	0.354794	newly	361.	304.48	{212., 411.}	21.5789	0.111538	Reff	0.975724	0.915293	{0.893358, 0.933379}	6.1934	ar	4667.	3663.84	{3232., 4125.}	21.4948
31	R0	{1.16062, 4.47529, 0.1}	{1.25635, 3.40894, 0.02}	{{1.22544, 3.07157, 0.}, {1.28596, 3.98513, 0.}}	{8.24816, 23.8275, 118.}	inf	237.	214.833	{140.522, 282.497}	19.3667	0.0987976	newly	360.	462.38	{330., 579.}	34.7444	0.127874	Reff	0.985599	1.06962	{1.04437, 1.09268}	8.5249	ar	3037.	3810.52	{3191., 4244.}	25.4949
32	R0	{1.37647, 18.7122, 2.}	{1.21663, 7.00826, 2.92}	{{1.21159, 3.57392, 1.}, {1.21725, 8.76728, 4.}}	{11.6123, 62.5471, 88.}	inf	357.	153.96	{60.9012, 209.351}	56.874	0.414965	newly	145.	146.46	{115., 172.}	14.731	0.0688319	Reff	1.02478	0.930481	{0.92413, 0.938658}	9.20209	ar	4824.	3303.7	{2719., 3756.}	31.5153
33	R0	{1.35084, 6.08841, 0.1}	{1.29001, 3.96435, 0.14}	{{1.25474, 3.26445, 0.}, {1.28596, 3.98513, 0.}}	{6.13626, 39.4806, 222.}	inf	485.	307.263	{208.26, 362.252}	42.7421	0.239227	newly	533.	496.88	{393., 607.}	13.3959	0.0626636	Reff	0.972335	0.950365	{0.928397, 0.95728}	3.39473	ar	4759.	4206.54	{3702., 4546.}	14.4018
34	R0	{1.36912, 6.316, 1.}	{1.24597, 2.57387, 0.}	{{1.24751, 2.5364, 0.}, {1.24751, 2.5364, 0.}}	{8.995, 59.2485, 100.}	inf	294.	137.224	{55.5956, 209.906}	53.325	0.390735	newly	335.	356.02	{149., 528.}	33.4925	0.162485	Reff	1.06983	0.993138	{0.981705, 1.00365}	7.16864	ar	4537.	3298.66	{2752., 3816.}	27.2942
35	R0	{1.35831, 7.09038, 1.}	{1.25954, 3.38605, 0.}	{{1.22544, 2.5364, 0.}, {1.28596, 3.98513, 0.}}	{7.27159, 52.2444, 100.}	inf	375.	197.223	{125.467, 259.76}	47.4072	0.295857	newly	358.	363.28	{263., 464.}	18.2514	0.0828249	Reff	0.949459	0.902755	{0.884947, 0.919204}	4.91901	ar	4667.	3813.07	{3433., 4174.}	18.2972
36	R0	{1.28315, 5.89938, 0.1}	{1.24772, 2.61263, 0.}	{{1.24751, 2.5364, 0.}, {1.24751, 3.07157, 0.}}	{2.76103, 55.7135, 100.}	inf	365.	179.886	{103.801, 247.51}	50.7161	0.327156	newly	398.	425.86	{264., 573.}	24.7638	0.105764	Reff	0.92297	0.920583	{0.911993, 0.9294}	0.630381	ar	4460.	3656.76	{3305., 3973.}	18.0099
37	R0	{1.19303, 2.75113, 0.1}	{1.24714, 2.55439, 0.}	{{1.24751, 2.5364, 0.}, {1.24751, 2.5364, 0.}}	{4.53582, 7.99038, 100.}	inf	171.	187.69	{94.4319, 262.35}	32.6566	0.142841	newly	357.	511.39	{272., 724.}	54.5294	0.188447	Reff	0.971365	1.01334	{1.0034, 1.02528}	4.32175	ar	3142.	3556.08	{2978., 4001.}	15.6537
38	R0	{1.36033, 7.8088, 1.}	{1.25194, 3.28211, 0.}	{{1.22544, 3.07157, 0.}, {1.25474, 3.26445, 0.}}	{7.96769, 57.9691, 100.}	inf	377.	177.749	{107.578, 236.121}	52.8517	0.344554	newly	348.	358.71	{239., 480.}	22.7443	0.0999182	Reff	1.02419	0.967518	{0.95039, 0.980187}	5.53355	ar	4941.	3516.32	{3090., 3982.}	28.8338
39	R0	{1.27353, 3.70845, 0.1}	{1.24779, 2.61991, 0.}	{{1.24751, 2.5364, 0.}, {1.24751, 3.07157, 0.}}	{2.02083, 29.3529, 100.}	inf	255.	190.965	{98.0833, 275.046}	29.1233	0.18014	newly	460.	453.19	{246., 636.}	27.4674	0.137596	Reff	0.944322	0.933226	{0.92253, 0.944401}	1.25932	ar	3725.	3627.86	{3099., 4029.}	7.7745
40	R0	{1.19983, 12.1486, 0.1}	{1.17082, 5.77111, 1.}	{{1.17082, 5.77111, 1.}, {1.17082, 5.77111, 1.}}	{2.41747, 52.4957, 900.}	inf	151.	88.7149	{68.2156, 108.645}	41.2484	0.238179	newly	79.	103.97	{78., 130.}	34.1899	0.123182	Reff	0.976302	0.959992	{0.957599, 0.962356}	1.67052	ar	3619.	2649.02	{2229., 3057.}	26.8024
41	R0	{1.20935, 2.67169, 0.1}	{1.24751, 2.5364, 0.}	{{1.24751, 2.5364, 0.}, {1.24751, 2.5364, 0.}}	{3.15566, 5.06367, 100.}	inf	236.	215.159	{123.544, 311.66}	25.4604	0.12942	newly	573.	550.13	{339., 813.}	26.1309	0.125978	Reff	0.945228	0.977656	{0.965209, 0.988333}	3.43125	ar	3429.	3652.81	{3160., 4087.}	10.59
42	R0	{1.23313, 4.74781, 1.}	{1.24123, 3.31462, 0.18}	{{1.21159, 3.07157, 0.}, {1.28596, 3.98513, 1.}}	{1.61042, 30.1863, 88.}	inf	192.	151.169	{96.8303, 195.639}	23.6514	0.130055	newly	278.	310.09	{188., 428.}	26.3201	0.108768	Reff	0.975036	0.986479	{0.966293, 1.0147}	1.58675	ar	3835.	3361.42	{2855., 3884.}	13.2772
43	R0	{1.26105, 2.39713, 0.1}	{1.24751, 2.5364, 0.}	{{1.24751, 2.5364, 0.}, {1.24751, 2.5364, 0.}}	{1.07347, 5.81005, 100.}	inf	151.	198.604	{109.131, 277.341}	42.8531	0.156899	newly	396.	490.66	{298., 676.}	37.9444	0.147015	Reff	0.95865	0.942421	{0.93221, 0.9523}	1.6998	ar	3723.	3658.71	{3254., 4007.}	6.89632
44	R0	{1.36948, 8.88006, 0.1}	{1.22528, 3.27155, 0.33}	{{1.21159, 3.07157, 0.}, {1.25474, 3.57392, 1.}}	{10.5293, 63.1584, 364.}	inf	376.	145.92	{81.3148, 201.854}	61.1916	0.437453	newly	309.	298.56	{180., 404.}	23.8576	0.117275	Reff	1.06984	0.985376	{0.969753, 1.00186}	7.89481	ar	4437.	3247.12	{2799., 3688.}	26.8172
45	R0	{1.34882, 3.3791, 0.1}	{1.42794, 3.8173, 0.}	{{1.24751, 2.5364, 0.}, {1.47488, 4.06661, 0.}}	{7.8184, 19.4519, 100.}	inf	365.	455.277	{320.505, 578.725}	30.3711	0.113661	newly	717.	871.91	{681., 1060.}	24.2831	0.0916748	Reff	1.04641	1.09448	{0.98058, 1.12651}	6.34726	ar	4422.	5244.79	{4149., 5733.}	21.2897
46	R0	{1.23774, 4.34917, 0.1}	{1.24952, 3.20115, 0.01}	{{1.22544, 2.5364, 0.}, {1.28596, 3.98513, 0.}}	{1.59068, 26.3963, 108.}	inf	261.	184.887	{107.643, 244.653}	30.8586	0.18713	newly	370.	401.81	{257., 525.}	25.4027	0.112146	Reff	0.996752	1.01572	{0.993902, 1.03887}	2.05054	ar	3685.	3567.33	{2980., 4086.}	9.04885
47	R0	{1.34327, 4.78811, 0.1}	{1.41594, 5.40191, 0.61}	{{1.22544, 3.07157, 0.}, {1.94282, 12.1446, 2.}}	{15.0379, 58.0005, 652.}	inf	281.	426.701	{161.192, 994.557}	77.3219	0.225443	newly	448.	551.91	{355., 689.}	32.8147	0.127206	Reff	1.0953	1.12577	{1.01354, 1.44314}	11.8116	ar	4517.	4809.82	{3406., 7780.}	28.9604
48	R0	{1.24366, 4.25814, 0.1}	{1.24689, 2.5511, 0.03}	{{1.24751, 2.5364, 0.}, {1.24751, 2.5364, 0.}}	{0.370912, 40.0889, 128.}	inf	209.	161.447	{80.9375, 231.798}	30.3661	0.179416	newly	310.	421.88	{232., 599.}	48.4581	0.175989	Reff	0.987217	0.995707	{0.987243, 1.00572}	1.0547	ar	3678.	3429.19	{2774., 3896.}	10.053
49	R0	{1.36017, 7.5415, 1.}	{1.21109, 2.9748, 0.05}	{{1.06738, 2.19254, 0.}, {1.24751, 3.26445, 0.}}	{10.9602, 60.5543, 97.}	inf	190.	107.517	{49.4818, 168.374}	43.6442	0.294619	newly	183.	251.12	{123., 405.}	53.5847	0.189988	Reff	1.12513	1.01172	{0.900714, 1.03593}	10.0799	ar	4178.	2947.04	{2093., 3574.}	29.4629
50	R0	{1.25333, 6.63578, 1.}	{1.22811, 3.0585, 0.04}	{{1.22544, 3.07157, 0.}, {1.24751, 3.07157, 0.}}	{2.02313, 53.909, 96.}	inf	193.	110.99	{61.4143, 154.24}	42.4921	0.267196	newly	204.	230.6	{136., 318.}	31.0196	0.12918	Reff	0.945763	0.93681	{0.92861, 0.951491}	1.19009	ar	3401.	3200.51	{2843., 3518.}	8.34313
